# Plastid proteome prediction for diatoms and other algae with secondary plastids of the red lineage

**DOI:** 10.1111/tpj.12734

**Published:** 2015-01-06

**Authors:** Ansgar Gruber, Gabrielle Rocap, Peter G Kroth, E Virginia Armbrust, Thomas Mock

**Affiliations:** 1 Fachbereich Biologie, Universität KonstanzKonstanz, 78457, Germany; 2 School of Oceanography, Center for Environmental Genomics, University of WashingtonSeattle, WA, 98195, USA; 3 School of Environmental Sciences, University of East AngliaNorwich Research Park, NR4 7TJ, Norwich, UK

**Keywords:** *Thalassiosira pseudonana*, *Phaeodactylum tricornutum*, chloroplast, proteome, prediction, technical advance

## Abstract

The plastids of ecologically and economically important algae from phyla such as stramenopiles, dinoflagellates and cryptophytes were acquired via a secondary endosymbiosis and are surrounded by three or four membranes. Nuclear-encoded plastid-localized proteins contain N-terminal bipartite targeting peptides with the conserved amino acid sequence motif ‘ASAFAP’. Here we identify the plastid proteomes of two diatoms, *Thalassiosira pseudonana* and *Phaeodactylum tricornutum*, using a customized prediction tool (ASAFind) that identifies nuclear-encoded plastid proteins in algae with secondary plastids of the red lineage based on the output of SignalP and the identification of conserved ‘ASAFAP’ motifs and transit peptides. We tested ASAFind against a large reference dataset of diatom proteins with experimentally confirmed subcellular localization and found that the tool accurately identified plastid-localized proteins with both high sensitivity and high specificity. To identify nucleus-encoded plastid proteins of *T. pseudonana* and *P. tricornutum* we generated optimized sets of gene models for both whole genomes, to increase the percentage of full-length proteins compared with previous assembly model sets. ASAFind applied to these optimized sets revealed that about 8% of the proteins encoded in their nuclear genomes were predicted to be plastid localized and therefore represent the putative plastid proteomes of these algae.

## Introduction

Plastids arose through endosymbiotic processes – a primary endosymbiosis of a cyanobacterium gave rise to red and green algae and the subsequent evolution of plants, and multiple secondary endosymbioses of either a red or a green alga gave rise to a broad diversity of eukaryotic microbes. Marine microalgae with secondary plastids from the red lineage contribute significantly to global biogeochemical cycles and support productive marine food webs (Cavalier-Smith, 1999). Major groups include diatoms, coccolithophores, cryptophytes, dinoflagellates, and apicomplexans. Plastids in these organisms have a complex structure with either three or four membranes, most with the endoplasmic reticulum (ER) as the outermost membrane (Kroth, 2002). The majority of genes from the original endosymbionts were either lost, replaced by genes of the host or transferred to the nucleus of the host; only a minority of the genes was retained on the original endosymbiont genome (Timmis *et al*., 2004). Of those organisms with a secondary plastid of the red lineage, only cryptophytes possess a remnant nucleus from the endosymbiont – the nucleomorph, which is located in the periplastidic space between the second and third envelope membrane (Curtis *et al*., 2012).

Consequently, the majority of proteins required for plastid function is encoded in the nucleus and subsequently transported to the plastid. Delivery of nuclear-encoded plastid proteins across multiple membranes requires an efficient protein import system (Gruber *et al*., 2007), which includes protein transport via the ER. All known nuclear-encoded plastid-localized proteins in cells with secondary plastids of the red lineage possess bipartite N-terminal pre-sequences that consist of an ER-type signal peptide followed immediately by a transit peptide (Kroth, 2002; Patron and Waller, 2007). The transit peptide is cleaved off the mature protein upon completion of the import reaction, likely by a specific stromal processing peptidase (Huesgen *et al*., 2013).

The transit peptide domains of bipartite plastid targeting pre-sequences commonly begin with a phenylalanine residue at the +1 position after the signal peptide cleavage site (Kroth, 2002; Armbrust *et al*., 2004; Patron and Waller, 2007), which is crucial for successful plastid protein import (Apt *et al*., 2002; Kilian and Kroth, 2005; Gruber *et al*., 2007). The transit peptide contains a high proportion of hydroxylated residues, few negatively charged residues, and a net positive charge (Patron and Waller, 2007), which is also necessary for plastid protein import (Felsner *et al*., 2010). Other features of the transit peptide, including its length, are less critical for plastid import (Apt *et al*., 2002; Kilian and Kroth, 2005; Gruber *et al*., 2007). The phenylalanine at the +1 position of the transit peptide is part of a conserved sequence motif (‘ASAFAP’ motif) surrounding the signal peptide cleavage site in diatoms (Kilian and Kroth, 2005; Gruber *et al*., 2007), cryptophytes (Gould *et al*., 2006a; Patron and Waller, 2007), and dinoflagellates (Patron *et al*., 2005; Patron and Waller, 2007). This distinctive motif is a good marker for identifying nuclear-encoded plastid proteins based on DNA sequence data (Gruber *et al*., 2007; Gruber and Kroth, 2014).

Here we present the results of a genomewide prediction of nucleus-encoded plastid proteins for the diatoms *Thalassiosira pseudonana* and *Phaeodactylum tricornutum*, based on recognition of ASFAP motifs, combined with a composition-based evaluation of the transit peptide downstream of the cleavage site.

## Results and discussion

### Characterization of the ‘ASAFAP’ motif

The plastid protein prediction was initiated with a set of putative plastid-targeted proteins from the diatoms *Thalassiosira pseudonana* and *Phaeodactylum tricornutum*, compiled based on the lists of nucleus-encoded and plastid-targeted proteins published by Armbrust *et al*. (2004) (for *T. pseudonana*), Gruber *et al*. (2007) (for *P. tricornutum*), and Kroth *et al*. (2008) (*P. tricornutum* and *T. pseudonana*). These proteins were supplemented with additional proteins that are most likely plastid-targeted based on functional annotation. Furthermore, homologues of proteins from the *T. pseudonana* lists were searched in the *P. tricornutum* genome and vice versa.

For maximum consistency between the sequence sets for the two species, all sequences found in only one of the organisms were removed from the set. To avoid potential overfitting of the data, we reduced the level of homology within the protein set using an ‘all against all’ BLAST search of the candidate sequences from *T. pseudonana* and *P. tricornutum*. Only sequences that paired with one homologue from the other diatom species (instead of a sequence from the same species) were retained to minimize inclusion of gene duplications present in only one organism. The highest level of homology present in the sequence set therefore corresponds to the time of independent evolution since the split between the pennate and centric diatom lineages, which took place approximately 90 million years ago (Bowler *et al*., 2008). The final set consists of 83 orthologous pairs of putative plastid-targeted protein sequences from *T. pseudonana* and *P. tricornutum* (Table S1).

Via proteomic amino-termini profiling, Huesgen *et al*. (2013) recently identified 1295 unique N-terminus peptides from 939 nuclear-encoded *T. pseudonana* proteins. These N-terminal peptides in many cases represent N-termini of native functional proteins, after cleavage of N-terminal targeting signals. The peptide list also contains N-termini of proteins that are not processed *in vivo*, as well as the products of internally cleaved proteins. Searched against our *T. pseudonana* dataset, 44 of the N-terminal peptides identified by Huesgen *et al*. (2013) match 36 of the 83 *T. pseudonana* sequences (Table S1). For 31 of the matches the position of our manually identified signal peptide cleavage site lies between 14 and 95 amino acid residues upstream of the N-terminal peptide, and therefore supports the presence of a transit peptide-like domain that is actually cleaved off. Three of the peptides match the transit peptide domain itself with the peptide starting with the +1 position of the predicted cleavage site or one position further downstream, which supports that the signal peptides as well as transit peptides are cleaved off independently from each other. It should be noted that in one case (*T. pseudonana* protein ID 270231) there is N-terminal peptide support for both the N-terminus of the transit peptide-like domain with the signal peptide cleaved off as well as for the N-terminus of the putative mature protein after cleavage of a 12-residue transit peptide. Ten of the peptides correspond to N-termini derived from internal cleavage of the protein after 133–1242 residues (Table S1).

**Figure 1:**
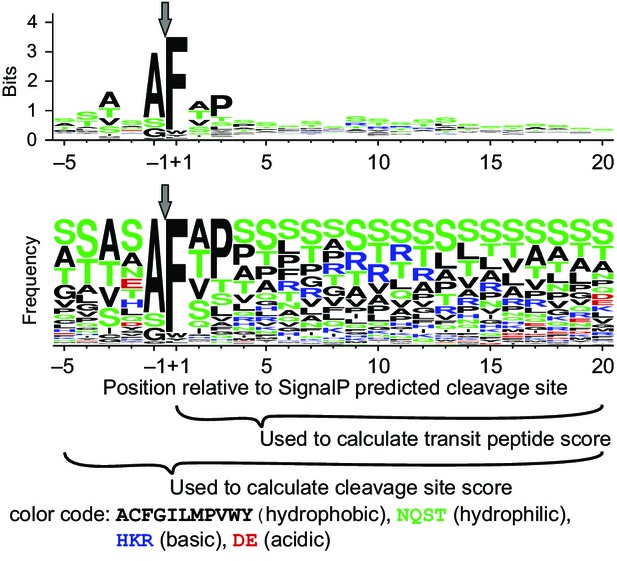
Conserved cleavage site motif.Conserved sequence motif surrounding the signal peptide cleavage site for 166 putative plastid-targeted protein sequences from *T. pseudonana* and *P. tricornutum* (see Table S1). Sequence logos represent the scoring matrix used to calculate cleavage site and transit peptide scores for SignalP positive sequences. Sequence logos and frequency plots (Schneider and Stephens, 1990) were created with WebLogo (Crooks *et al*., 2004; http://weblogo.berkeley.edu/).

Despite the overall divergence between *T. pseudonana* and *P. tricornutum* (Bowler *et al*., 2008), the plastid targeting motifs are similar in both organisms, and also similar to the N-terminal signal found in the list of 63 *T. pseudonana* transit peptides published by Huesgen *et al*. (2013) (Figure S1). We therefore combined the sequence sets from *T. pseudonana* and *P. tricornutum* to generate a scoring matrix (Table S2) based on the frequency of occurrence of each amino acid weighted by the amount of information at each position in the sequence logo (Figure1). This was used to develop a single plastid protein prediction method.

### Prediction of signal peptides and cleavage sites

The signal peptide of plastid-targeted proteins in diatoms and other organisms with secondary plastids can be identified via the prediction program SignalP (Nielsen *et al*., 1997; Emanuelsson *et al*., 2007) that has been developed through a number of versions. The most current versions are SignalP 3.0 (Bendtsen *et al*., 2004) and SignalP 4.1 (Petersen *et al*., 2011). SignalP 3.0 employs either a neuronal network (NN) or a hidden Markov (HMM) model to identify the signal peptide (Nielsen and Krogh, 1998; Bendtsen *et al*., 2004), SignalP 4.1 exclusively uses a NN, but can be adjusted to two levels of sensitivity (Petersen *et al*., 2011). SignalP 3.0 NN recognized a signal peptide in 163 of the 166 test proteins (83 sequences from *T. pseudonana* and *P. tricornutum* each, see Table S1) whereas SignalP 3.0 HMM recognized 165 signal peptides (Table S1). SignalP 4.1 identified signal peptides in 141 or 161 of the 166 sequences, depending on the choice of sensitivity (Table S1).

In diatoms, the signal peptide cleavage site is spanned by the ‘ASAFAP’ motif (Kilian and Kroth, 2005; Gruber *et al*., 2007). In a comparison between manually identified cleavage site motifs with the predictions of the different SignalP variants, we found that the SignalP 3.0 NN prediction identified the ‘ASAFAP’ motif in 150 out of the 166 proteins, whereas the SignalP 3.0 HMM prediction identified the motif in 139 of the 166 proteins. SignalP 4.1, identified 148 of the cleavage site motifs (Table S1), the cleavage site predictions are identical for both sensitivity settings. For most of the tested sequences, cleavage site predictions are identical between the different SignalP versions; deviant predictions are found for all versions, with no particular overlap that would allow conclusions on the presence of non-canonical sequences in our set (Figure S2). Based on the highest level of congruency with the manual motif identifications, we decided to use the NN prediction of SignalP 3.0 for all subsequent analyses, and to additionally evaluate methods to increase the accuracy of the cleavage site predictions via the direct detection of ‘ASAFAP’ motifs.

**Figure 2:**
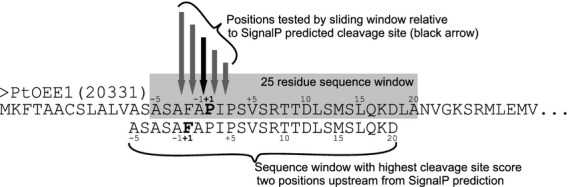
Sliding window and cleavage site score calculation.Conserved cleavage site motifs that differ from the SignalP predicted cleavage site are identified by calculating cleavage site scores for 25-position sequence windows surrounding the SignalP predicted cleavage site as shown on the example of the *Phaeodactylum tricornutum* oxygen evolving enhancer 1 (PtOEE1, GenBank AY191862, Protein ID 20331). See also Gruber *et al*. (2007) and Kilian and Kroth (2005) for detailed mutational analyses of this sequence.

For this, we used the information in the sequence logo to evaluate potential alternate cleavage sites. Because the highest bit scores within the sequence logo, and thus the greatest discriminating potential, were found on either side of the signal peptide cleavage site (Figure1), proteins were first scored over a 25 amino acid sequence window from −5 to +20 around the SignalP 3.0 NN-predicted signal peptide cleavage site, using the scoring matrix (Table S2) generated from the 166 putative plastid-targeted proteins (Table S1). Next, proteins were scored over a sliding window of five residues, including two positions upstream and downstream of the SignalP predicted cleavage site (Figure2). The ASAFind predicted cleavage site corresponds to the cleavage site position with the highest score.

A signal peptide cleavage site is common to all ER-targeted proteins, including those that are targeted to the ER but not to the plastid. To discriminate between plastid proteins and other secretory proteins, we calculated a transit peptide score, again via the weighted scoring matrix, based on the 20 residues downstream of the signal peptide cleavage site. Thus, this transit peptide score does not evaluate the ER cleavage site itself.

### Plastid protein prediction

Because of the general trade-off between sensitivity (the ability to recognize true positives) and specificity (the ability to recognize true negatives), we opted to develop a plastid protein prediction protocol at two confidence levels, tuned for either high sensitivity or high specificity. For the statistical evaluation of our prediction method we compiled a set of reference proteins based on available experimental protein location data (e.g. fusion of reporter genes, proteomic studies or immuno-electron microscopy) for *P. tricornutum* proteins (Table S3). This dataset included plastid-targeted protein sequences, as well as proteins targeted to other compartments such as the ER and mitochondria. It consisted of 132 proteins, 19 of which were, by coincidence, also included in the sequence set used to calculate the initial sequence logos. The use of largely separate sequence sets for generating the scoring matrix and evaluating the prediction ensures that the reference data are not overfit. Sequences in the reference set were classified as positive if they were experimentally shown to be plastid targeted or as negative if they were experimentally shown to be targeted to another compartment (Table S3).

**Figure 3:**
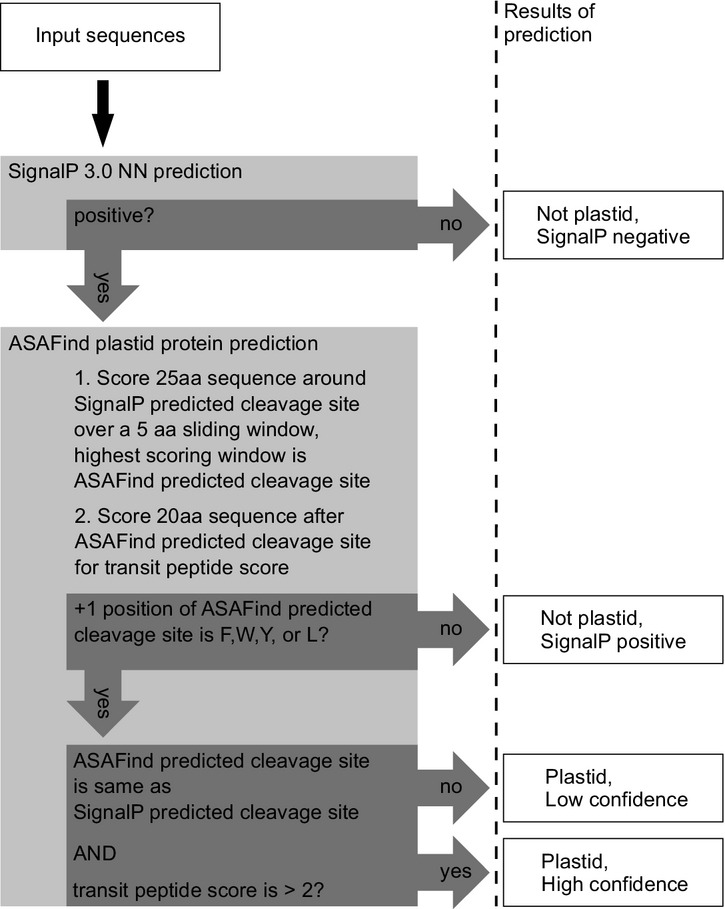
Plastid protein prediction.Decision making steps for the prediction of plastid proteins, the ASAFind prediction is based on the results of the signal peptide prediction via SignalP 3.0 neuronal networks (Nielsen and Krogh, 1998; Bendtsen *et al*., 2004; Emanuelsson *et al*., 2007).

Plastid-targeted reference proteins were best distinguished from non-plastid-targeted reference proteins by the following protocol (Figure3). If the SignalP 3.0 NN prediction was negative, the sequence was defined as ‘not plastid, SignalP negative.’ If the SignalP 3.0 NN prediction was positive, the window spanning two positions each upstream and downstream of the SignalP NN-identified cleavage site was further evaluated to identify the position with the highest cleavage site score. This position was deemed the ASAFind predicted cleavage site. If the first amino acid of the ASAFind predicted transit peptide was an amino acid other than ‘F’, ‘W’, ‘Y’ or ‘L’, the sequence was defined as ‘not plastid, SignalP positive’. These proteins are candidates for retention in the ER or for other targeting via the secretory system. If an ‘F’, ‘W’, ‘Y’ or ‘L’ residue was present at the first position of the ASAFind predicted transit peptide, the sequence was classified as potentially plastid targeted and evaluated further. If the ASAFind predicted cleavage site coincided with the SignalP prediction and the transit peptide score was higher than 2, the sequence was defined as ‘plastid, high confidence’, otherwise the sequence was defined as ‘plastid, low confidence’ (Figure3).

This protocol was optimized with our reference set as the gold standard. We empirically tested the performance of different prediction approaches and parameters (such as sliding window ranges or score cut-offs) by calculating sensitivity, specificity and Matthews correlation coefficients (MCC) or by receiver operating characteristics (ROC) plot analyses (Baldi *et al*., 2000; Brown and Davis, 2006; Fawcett, 2006).

**Figure 4:**
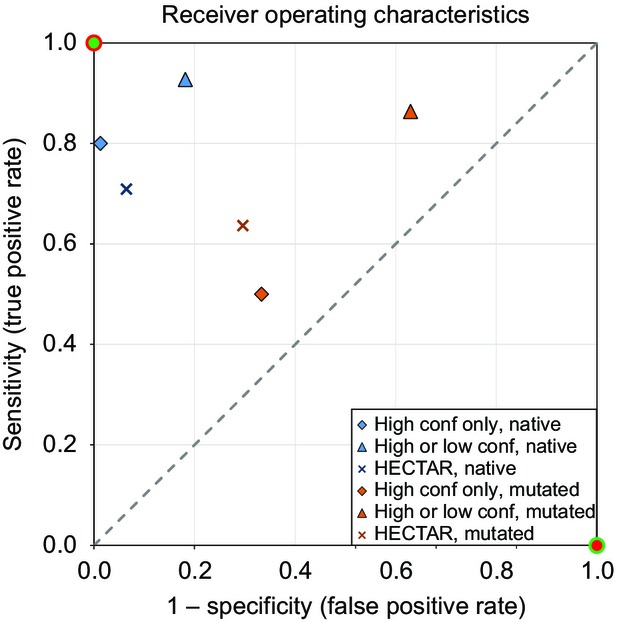
Receiver operating characteristics.Receiver operating characteristics (ROC) plot of plastid protein prediction methods evaluated with sets of native (Tables S3 and S4) or mutated (Table S5) reference sequences, see Table S6 for additional counts/scores. ‘High conf only, native’ are high confidence only predictions tested with the native experimental reference set; ‘high or low conf, native’ are high or low confidence predictions tested with the native experimental reference set; ‘HECTAR, native’ are predictions by HECTAR (Gschloessl *et al*., 2008) tested with the native experimental reference set (Tables S3 and S4); ‘high conf only, mutated’ are high confidence only predictions tested with the mutated sequences reference set (Table S5); ‘high or low conf, mutated’ are high or low confidence predictions tested with the mutated sequences reference set; ‘HECTAR, mutated’ are predictions by HECTAR (Gschloessl *et al*., 2008) tested with the mutated sequences reference set. Hypothetical ‘perfect’ (green dot with red ring) and ‘perfect inverse’ (red dot with green ring) predictions are included on the diagram; the dashed line corresponds to random guesses.

Based on these analyses, the ‘plastid, low confidence’ prediction is highly sensitive, while the ‘plastid, high confidence’ prediction is extremely specific (Figure4). The MCC for our method is higher (Table1) than for the specialized prediction server HECTAR (Gschloessl *et al*., 2008) which combines a number of publically available subcellular localization methods using a Support Vector Machine to produce a prediction. The increase in prediction performance of ASAFind is mainly driven by the enhanced sensitivity of our approach; as a consequence, HECTAR should be used with care when a high sensitivity is desired.

**Table 1:** Plastid protein prediction statistics. Performance of the plastid protein prediction methods evaluated with *Phaeodactylum tricornutum* sequences of proteins with experimentally determined intracellular location (see Table S3). See Table S6 for formulas and additional counts/scores, MCC: Matthews correlation coefficient

	High confidence only	High or low confidence	HECTAR^a^
Sensitivity	0.80	0.93	0.71
Specificity	0.99	0.82	0.94
MCC	0.82	0.74	0.67

a See Gschloessl *et al*. (2008).

In addition to the reference protein set, we also collected 49 sequences that were mutated in previous studies to pinpoint the crucial components of the targeting signal (Kilian and Kroth, 2005; Gruber *et al*., 2007; Felsner *et al*., 2010) (Table S5). As expected, the prediction methods performed considerably worse with this mutated protein test set (Figure4 and Tables S4–S6), emphasizing that native targeting pre-sequences are under strong selection pressures to maintain their functionality. This result shows that experimentally engineered pre-sequences are useful for the characterization of the exact requirements for the targeting signal as performed by Apt *et al*. (2002), Felsner *et al*. (2010), Gruber *et al*. (2007) or Kilian and Kroth (2005), however, due to the artificial nature of these sequences, they are of limited use as templates for the development of prediction algorithms.

The efficiency of our method depends on the presence of the conserved ‘ASAFAP’ motif. Mernberger *et al*. (2014) recently developed a motif-independent method for subcellular localization of proteins, with the stated goal of predicting plastid proteins in organisms with limited information on potential protein localization signals. Although their methods were tested with the diatom *P. tricornutum*, we were unable to compare the performance of their methods with ours because neither the sequences used in their reference set nor the performance metrics are specified in the manuscript and the method is not available for public use. Mernberger *et al*. (2014) do not compare the performance of their prediction methods to the performance of the dedicated prediction tool HECTAR (Gschloessl *et al*., 2008), but do compare to other established methods including TargetP (Emanuelsson *et al*., 2000), WoLF PSORT (Horton *et al*., 2007) and MultiLoc2 (Blum *et al*., 2009), none of which was developed to predict protein localization in secondary plastids. Therefore it remains unclear whether the apparent advantage of Mernberger *et al*.'s (2014) approach over the other tested prediction tools comes from a methodological improvement or from use of specific training sets for the tested organisms.

The performance of our method was also evaluated in the cryptophyte *Guillardia theta* with 54 homologues of proteins from the *P. tricornutum* reference set (Curtis *et al*., 2012). For *G. theta*, the ‘plastid, low confidence’ prediction has a sensitivity of 0.85 and a specificity of 0.88 (MCC: 0,73), while the ‘plastid, high confidence’ prediction has a sensitivity of 0.70 and a specificity of 0.97 (MCC: 0.72) (Curtis *et al*., 2012). Use of our diatom-optimized prediction method for other organisms with secondary plastids of the red algal lineage, such as cryptophytes, appears to come with a loss of sensitivity, at similar levels of specificity (compare to Table1).

### Gene catalog optimization

**Figure 5:**
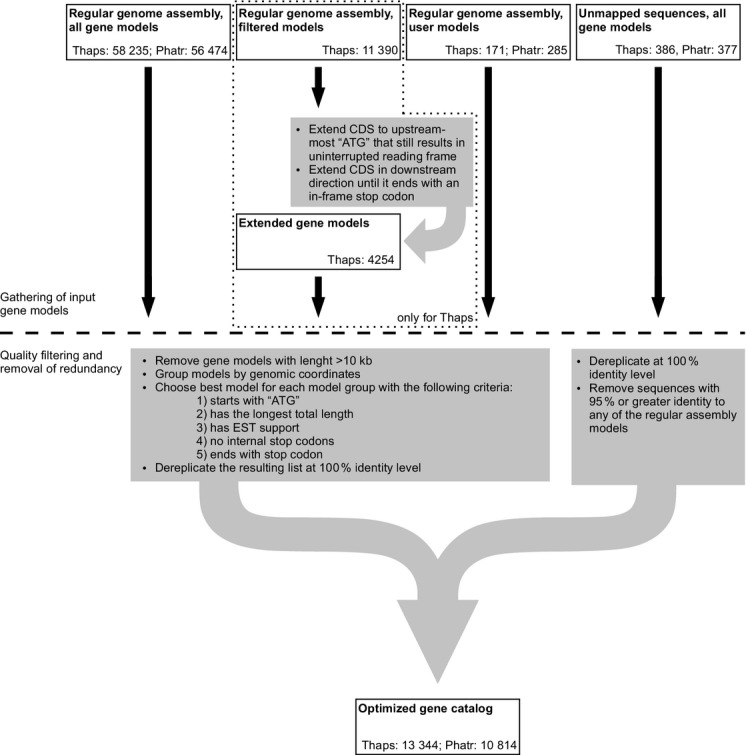
Gene catalog optimization.Source gene catalogs and procedures applied to obtain the optimized gene catalogs for *Thalassiosira pseudonana* (Thaps) and *Phaeodactylum tricornutum* (Phatr), see Datasets S1 and S2.

The predicted complete proteomes of *T. pseudonana* and *P. tricornutum* were optimized to ensure that the datasets used for plastid localization predictions were composed primarily of full-length proteins (Figure5). This was necessary because of the challenges involved in complete gene annotation, especially in non-model organisms, where identification of 5′ exons can be particularly problematic (Yandell and Ence, 2012; Gruber and Kroth, 2014). Indeed, many studies involving diatom protein annotation have resorted to manual extension of original gene models (Kroth *et al*., 2008; Huesgen *et al*., 2013). For our optimization, all protein predictions for the two diatoms available through the Joint Genome Institute genome portal (http://www.jgi.doe.gov) were considered (over 50 000 gene models for each genome), including user-defined gene models. The *T. pseudonana* models were extended in both directions–upstream to the first ‘ATG’ codon and downstream to the first stop codon in the same reading frame. For both diatoms, models over 10 kb in length were assumed to be incorrect and were excluded from the dataset. The resulting protein set for each diatom was further optimized by identifying the longest gene model for a given position on the genome with an N-terminal ATG (initiator methionine codon), with no internal stop codons, with EST support, and with a C-terminal stop codon. The so-called Joint Genome Institute (JGI) ‘filtered’ gene model was selected in cases in which multiple gene models with identical sequences fulfilled these criteria. Identical sequences were removed from the optimized set of gene models. As a final step, gene models derived from unknown chromosome locations were evaluated for inclusion in the optimized set. Proteins that were <95% identical to a protein derived from a known chromosome location were added to the optimized protein datasets for both diatoms (Datasets S1 and S2).

The resulting optimized datasets were composed of a larger set of predicted proteins than is currently available for download through the JGI genome portal (Table2 and Datasets S1 and S2). The percentage of gene models beginning with an ATG increased substantially in both genomes (from 83 to 96% in *T. pseudonana* and from 89 to 98% in *P. tricornutum*, see Table2). The entire dataset was analyzed via SignalP 3.0 NN. The number of predicted proteins with signal peptides also increased, from 12 to 22% of the total proteins in *T. pseudonana* and from 14 to 24% in *P. tricornutum* (Table2). Signal peptides, although not conserved directly on the sequence level, nevertheless have to fulfill structural requirements that are a function of their primary sequence (Patron and Waller, 2007). The increased number of predicted signal peptides in our improved gene catalog therefore indicates that the additional sequence regions represent coding sequence under actual selection pressure, as opposed to untranslated regions, that are not under selection pressure to maintain signal peptide features. We also compared the experimentally verified sequences of our reference proteins with either the optimized dataset or the original JGI dataset. In the JGI dataset, gene model translation start sites were identical to those of 77 of the 131 experimentally verified proteins used as our reference sequences. With our optimized dataset, gene model translation start sites were identical to those of 121 of the 132 reference proteins (Table S3). Together, these findings emphasize the enhanced quality of our optimized datasets and reiterate the difficulty of predicted targeting pre-sequences based solely on homology to proteins from closely related organisms.

**Table 2:** Gene catalog optimization. Results of the gene catalog optimization procedure in comparison with *Thalassiosira pseudonana* (Thaps) and *Phaeodactylum tricornutum* (Phatr) genome assemblies

	Gene models total	Gene models beginning with ‘ATG’ (%)	Gene models with signal peptides (%)
Thaps v3.0 initial release^a^	11 776	–	1384 (12)
Thaps v3.0 filtered models^b^	11 390	9477 (83.2)	2077 (18.24)
Thaps optimized catalog^c^	13 344^d^	12 756 (95.59)	2915^e^ (21.85)
Phatr v2.0 initial release^a^	10 402	–	1479 (14)
Phatr v2.0 filtered models^b^	10 025	8917 (88.94)	2070 (20.65)
Phatr optimized catalog^c^	10 814^f^	10 611 (98.12)	2648^g^ (24.49)

a See Bowler *et al*. (2008).

b Downloaded via the JGI genome portals on 8 August 2011.

c See Figure5 and text for details.

d All sequences in Dataset S1.

e SignalP positive sequences in Table S7.

f All sequences in Dataset S2.

g SignalP positive sequences in Table S8.

### Plastid proteome prediction

**Figure 6:**
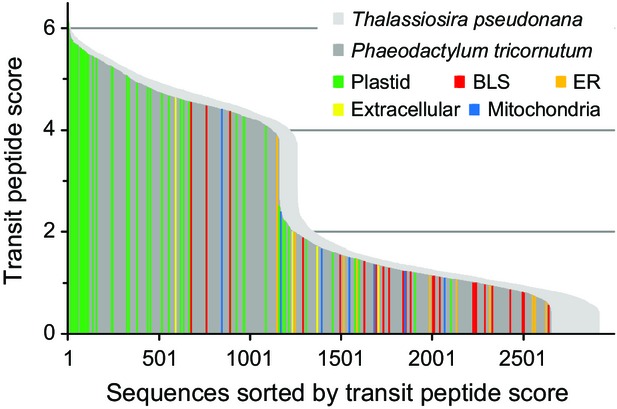
Transit peptide score distribution.Distribution of transit peptide scores for the sequence window with the highest cleavage site score of each SignalP positive sequence in the optimized gene catalogs, sequences from the native experimental reference set (Tables S3 and S4) are highlighted in the diagram for *Phaeodactylum tricornutum*, see Tables S7 and S8 for raw data. BLS, ‘blob’-like structure (Kilian and Kroth, 2005); ER, endoplasmic reticulum.

The optimized gene catalogs were used to identify nuclear-encoded plastid proteins in *T. pseudonana* (Table S7) and *P. tricornutum* (Table S8). The distribution of transit peptide scores is similar between *T. pseudonana* and *P. tricornutum* (Figure6). Both curves are characterized by a sudden decrease in the transit peptide score coinciding with the absence of a phenylalanine residue at the first position of the scored transit peptide. Our transit peptide score cut-off of two was empirically optimized for prediction performance. Analysis of this larger dataset indicates that proteins exceeding this cut-off may lack a phenylalanine residue at the first position of the transit peptide. In these instances, the other amino acids within the scoring matrix window have to contribute much more to the overall score. The scores attained by sequences from the reference set show that a cut-off of two is sufficient to separate plastid-targeted sequences from sequences targeted to other compartments (Figure6).

The *T. pseudonana* optimized gene catalog contains 2915 proteins with predicted signal peptides and the *P. tricornutum* optimized gene catalog contains 2648 proteins with predicted signal peptides (Table2). In *T. pseudonana* 996 proteins are predicted to localize to the plastid with high confidence and 895 proteins are predicted to localize to the plastid in *P. tricornutum* with high confidence. The higher total number of genes in the *T. pseudonana* genome (13 344) compared with *P. tricornutum* (10 814) therefore is largely driven by proteins that are not targeted to the plastid (Table3). Using the ‘plastid, high confidence’ criteria, the *G. theta* genome contains 755 plastid proteins, at a much higher total number of genes (24 840) (Curtis *et al*., 2012), so also in this case the genome expansion is mainly driven by genes encoding non-plastid proteins.

**Table 3:** Plastid protein prediction results. Predicted plastid proteins in the optimized gene catalogs for *Thalassiosira pseudonana* and *Phaeodactylum tricornutum*, numbers in parentheses are percentages of total number of sequences in the optimized gene catalogs, for complete prediction results see Tables S7 and S8

	*Thalassiosira pseudonana*	*Phaeodactylum tricornutum*
Plastid, high confidence only	996 (7.46)	895 (8.28)
Plastid, high or low confidence	1568 (11.75)	1608 (14.87)
Not plastid, SignalP positive	1347 (10.09)	1040 (9.62)
Not plastid, SignalP negative	10 429 (78.15)	8166 (75.51)

## Conclusion

ASAFind combines high sensitivity with high specificity compared to previously published prediction tools (Gschloessl *et al*., 2008; Mernberger *et al*., 2014), and provides a powerful method for *in silico* prediction of plastid proteins in algae with secondary plastids of the red lineage. Furthermore, it allows the user to adjust predictions either in favour of sensitivity or specificity in order to enable the discovery of new plastid proteins (high sensitivity) or to validate sequences (high specificity) predicted by other approaches. We provide here the approximately 8% of proteins encoded in the nuclear genomes of diatoms (*T. pseudonana* and *P. tricorntum*) that are predicted to be plastid localized with high specificity and high confidence. This percentage is similar to predictions for the green alga *Chlamydomonas reinhardtii* (7% nuclear-encoded plastid proteins; Terashima *et al*., 2011).

## Experimental procedures

### Annotation of scoring matrix and reference sets

Experimental data were compiled from published studies (Liaud *et al*., 2000; Apt *et al*., 2002; Domergue *et al*., 2003; Kilian and Kroth, 2004, 2005; Kroth *et al*., 2005; Tanaka *et al*., 2005; Gould *et al*., 2006b; Gruber *et al*., 2007, 2009; Lepetit *et al*., 2007; Siaut *et al*., 2007; Sommer *et al*., 2007; Kitao *et al*., 2008; Ast *et al*., 2009; Hempel *et al*., 2009, 2010; Kitao and Matsuda, 2009; Weber *et al*., 2009; Bullmann *et al*., 2010; Felsner *et al*., 2010; Joshi-Deo *et al*., 2010; Allen *et al*., 2011, 2012; Bruckner *et al*., 2011; Grouneva *et al*., 2011; Moog *et al*., 2011; Tachibana *et al*., 2011; Vugrinec *et al*., 2011; Sturm *et al*., 2013). The gene models for the experimentally tested proteins were manually verified (Table S3). The *Thalassiosira pseudonana* v3.0 (Armbrust *et al*., 2004; Bowler *et al*., 2008) and *Phaeodactylum tricornutum* v2.0 (Bowler *et al*., 2008) genome databases were accessed online via the United States Department of Energy Joint Genome Institute (JGI) genome portal (http://genome.jgi-psf.org/) (Grigoriev *et al*., 2012) using TBLASTN and BLASTP (Altschul *et al*., 1997). If none of the automatically created gene models was complete, gene models were manually edited with the editing function of the JGI genome portal (Grigoriev *et al*., 2012). Local BLAST (Altschul *et al*., 1997) searches were performed using the program BioEdit (Hall, 1999).

### Software and scripting techniques

For the prediction of signal peptides SignalP v3.0b (Bendtsen *et al*., 2004) and SignalP4.0 (Petersen et al., 2011) were installed locally on a Linux system running Ubuntu. SignalP was invoked using the ‘euk’ option and proteins were judged SignalP positive based on the NN criterion.

For statistical analyses and formatting, data were processed using Perl scripts (Strawberry Perl for Windows – 5.12.3.0, http://strawberryperl.com/). Statistical figures and the ROC plot were prepared following the methods described in (Baldi *et al*., 2000; Brown and Davis, 2006; Fawcett, 2006). Sequence logos (Schneider and Stephens, 1990) were prepared using the WebLogo (Crooks *et al*., 2004) server (http://weblogo.berkeley.edu/).

ASAFind was implemented in Python 2.7 (https://www.python.org/) using Biopython v1.63 (Cock *et al*. 2009) and is available as a web server at http://rocaplab.ocean.washington.edu/tools/asafind. A standalone version, which offers the option of a custom weight matrix based on sequences of user interest is also available at the same location and as Appendix S1.

### Input data

Protein sequences were downloaded from the JGI genome portals for *Thalassiosira pseudonana* v3.0 (Armbrust *et al*., 2004; Bowler *et al*., 2008) and *Phaeodactylum tricornutum* v2.0 (Bowler *et al*., 2008) on Aug. 8, 2011 (All Models and User Models) and 5 October 2012 (unmapped models) and processed as described in the results section.

## Supporting Information

Figure S1 Conserved transit peptides.

Figure S2 Cleavage site motif identification.

Table S1 Initial sequence set.

Table S2 Scoring matrix.

Table S3 Reference set.

Table S4 Prediction results for native reference sequences.

Table S5 Prediction results for mutated reference sequences.

Table S6 Prediction statistics.

Table S7 Prediction results for *Thalassiosira pseudonana*.

Table S8 Prediction results for *Phaeodactylum tricornutum*.

Dataset S1 Optimized *Thalassiosira pseudonana* gene catalog.

Dataset S2 Optimized *Phaeodactylum tricornutum* gene catalog.

Appendix S1 ASAFind Python script.
