# Supplementary material for: Plastid proteome prediction for diatoms and other algae with secondary plastids of the red lineage
Source: Plant J. 2015 Jan 6;81(3):519–28. doi: 10.1111/tpj.12734 (PMC4329603; doi:10.1111/tpj.12734)
Supplement: Figure S1 — Conserved transit peptides. [file tpj0081-0519-sd1.pdf]

Thalassiosira pseudonana, 83 sequences

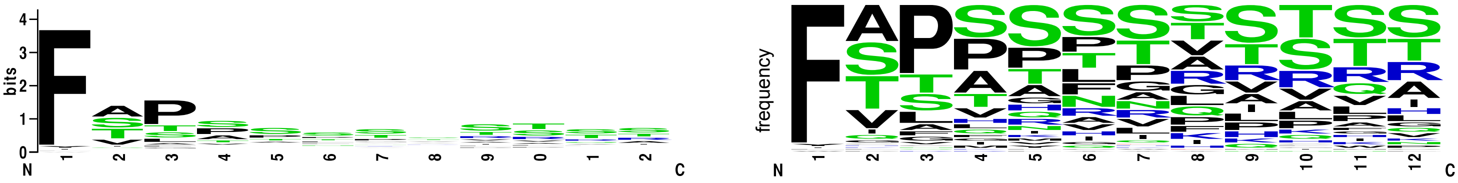

Phaeodactylum tricornutum, 83 sequences

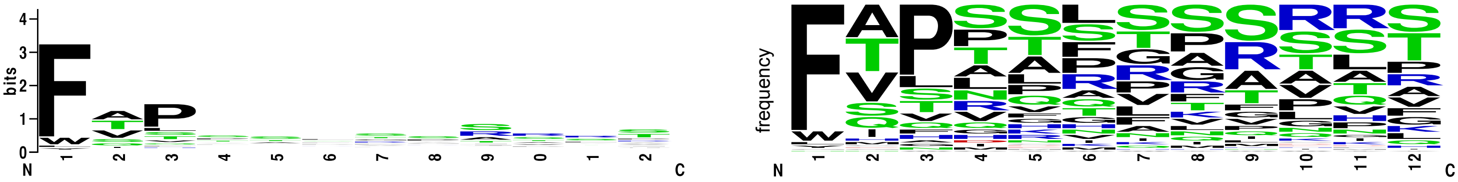

combined, 166 sequences

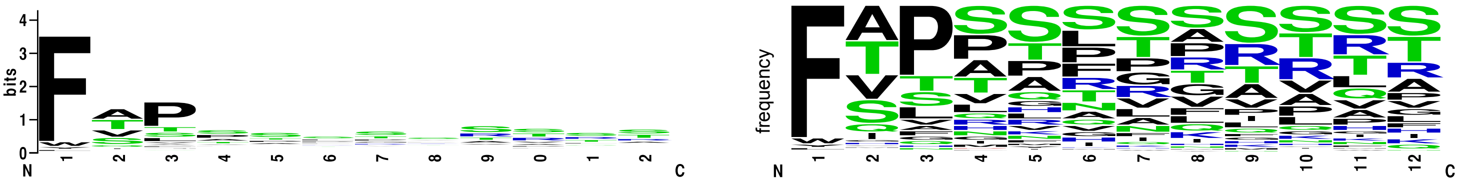

Thalassiosira pseudonana transit peptides from Huesgen et al. 2013, 63 sequences

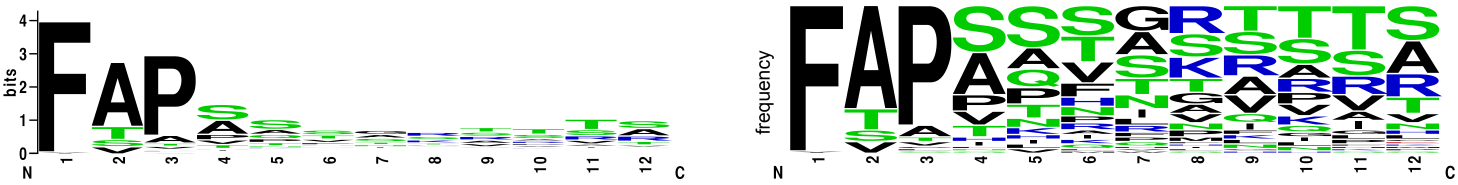

color code: **ACFGILMPVWY** (hydrophobic), **NQST** (hydrophilic), **HKR** (basic), **DE** (acidic)

Figure S1 (Gruber *et al.*, doi: 10.1111/tpj.12734)
