# Supplementary material for: Plastid proteome prediction for diatoms and other algae with secondary plastids of the red lineage
Source: Plant J. 2015 Jan 6;81(3):519–28. doi: 10.1111/tpj.12734 (PMC4329603; doi:10.1111/tpj.12734)
Supplement: Figure S2 — Cleavage site motif identification. [file tpj0081-0519-sd2.pdf]

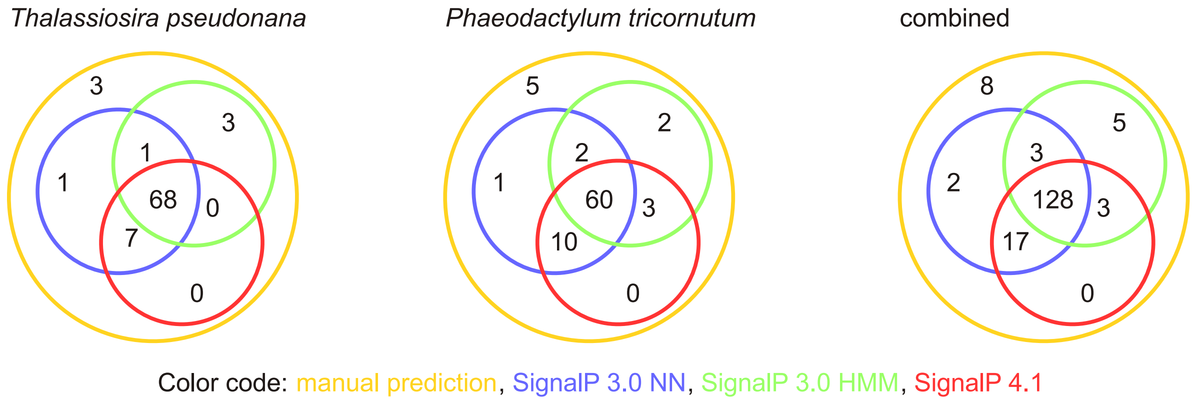

Figure S2 (Gruber *et al.*, doi: 10.1111/tpj.12734)
