# Supplementary material for: Plastid proteome prediction for diatoms and other algae with secondary plastids of the red lineage
Source: Plant J. 2015 Jan 6;81(3):519–28. doi: 10.1111/tpj.12734 (PMC4329603; doi:10.1111/tpj.12734)
Supplement: Table S3 — Reference set. [file tpj0081-0519-sd5.pdf]

Table S3[1]: Reference set. Proteins with experimentally determined intracellular location in *Phaeodactylum tricornutum*. Protein IDs refer to the U.S. Department of Energy Joint Genome Institute *Phaeodactylum tricornutum* v2.0 database (http://genome.jgi-psf.org/Phatr2/Phatr2.home.html) [6]; the 'Filtered models?' column indicates whether the filtered models contain a sequence with identical N-terminus (first 60 residues); the 'Optimized gene catalog?' column indicates whether the optimized gene catalog contains a sequence with identical N-terminus (first 60 residues); the 'Scoring matrix?' column indicates whether a sequence was used to build the matrix used to calculate the plastid score; 'Count' indicates whether the sequence was counted as positive or negative; the 'ASAFind, high or low confidence', 'ASAFind, high confidence only' and 'HECTAR' [16] columns indicate the classification of the sequence with each prediction method, see text for details. BLS, 'blob'-like structure [21]; FCP, fucoxanthin chlorophyll a/c-binding protein); FN, false negative; FP, false positive; ies, inter envelope space (compartment between the innermost and second innermost plastid membranes; N, negative; P, positive; Pl, plastid; PS, Photosystem; TN, true negative; TP, true positive.

Protein ID	Name	Location	Comment	Method	Filtered models?	Optimized gene catalog?	Scoring matrix?	Count	ASAFind, high or low confidence	ASAFind, high confidence only	HECTAR
17326	Lhl1/-RedCAP	Pl (thylakoid membrane, PS I)		two-dimensional BN/SDS-PAGE of thylakoid membranes followed by mass spectrometry [13] + GFP fusion [31]	yes	yes	no	P	TP	TP	TP
17388	salpha7-1	BLS		GFP fusion [28]	no	yes	no	N	TN	TN	TN
17531	unnamed	Pl (thylakoid membrane, PS I)	N-terminal extension possible, compare to protein ID 56750	two-dimensional BN/SDS-PAGE of thylakoid membranes followed by mass spectrometry [13]	yes	yes	no	P	TP	TP	TP
17683	SybA	ER		GFP fusion [28]	yes	no	no	N	TN	TN	TN
17766	Lhcr4	Pl (thylakoid membrane, PS I)	N-terminal extension possible, compare to protein ID 56749	two-dimensional BN/SDS-PAGE of thylakoid membranes followed by mass spectrometry [13]	yes	yes	no	P	TP	TP	TP
17972	PtSP1	Pl (ies)	supposedly enters the ies via the stroma (no experimental data on this process available)	self assembling GFP with MGD1 [8]	no	yes	no	P	TP	TP	TP
18049	Lhcf1/-FcpA	Pl (thylakoid membrane, FCP trimer)		two-dimensional BN/SDS-PAGE of thylakoid membranes followed by mass spectrometry [13] + mass spectrometry and westernblot after thylakoid membrane preparation [19]	yes	yes	yes	P	FN	FN	TP
18793	unnamed	Extracellular		mass spectrometric peptide mapping of extracellular proteins [7]	yes	yes	no	N	TN	TN	TN
19162	sCdc48-2	BLS		GFP fusion [28]	no	yes	no	N	TN	TN	TN
20030	CA-VIII	Mitochondria		GFP fusion [32]	no	no	no	N	TN	TN	TN
20331	OEE1	Pl		GFP fusion [20, 21, 14] + self-assembling GFP with AtpC [34]	yes	yes	no	P	TP	FN	TP

[1]Gruber *et al.*, doi: 10.1111/tpj.12734

Protein ID	Name	Location	Comment	Method	Filtered models?	Optimized gene catalog?	Scoring matrix?	Count	ASAFind, high or low confidence	ASAFind, high confidence only	HECTAR
20349	GLR/Gsr2	Mitochondria		GFP fusion [28]	no	yes	no	N	TN	TN	FP
20657	AtpC	Pl		GFP fusion [3, 20, 21, 11] + self-assembling GFP with OEE1 [34]	yes	yes	yes	P	TP	TP	TP
20779	FSA	Pl		GFP fusion [14]	yes	yes	yes	P	TP	TP	FN
21505	MGD1	Pl (ies)	supposedly enters the ies via the stroma (no experimental data on this process available), N-terminal extension possible, compare to protein ID 56712	GFP fusion and self-assembling GFP with PtSP1 [8]	no	no	no	P	TP	TP	TP
22006	Lhcf10	Pl (thylakoid membrane, FCP trimer)		two-dimensional BN/SDS-PAGE of thylakoid membranes followed by mass spectrometry [13]	yes	yes	no	P	TP	TP	TP
22110	sbeta7	BLS		GFP fusion [28]	no	yes	no	N	TN	TN	TN
22122	GapC1	Pl		immuno electron microscopy with GapC1 antibody [26]	yes	yes	yes	P	TP	TP	TP
22395	Lhcf8	Pl (thylakoid membrane, PS I + FCP trimer)		two-dimensional BN/SDS-PAGE of thylakoid membranes followed by mass spectrometry [13]	yes	yes	no	P	TP	TP	TP
22956	Lhcr2	Pl (thylakoid membrane, PS I)		two-dimensional BN/SDS-PAGE of thylakoid membranes followed by mass spectrometry [13]	yes	yes	yes	P	TP	TP	TP
22993	FbaC2	Pl		YFP fusion [2]	yes	yes	no	P	TP	TP	FN
23247	FBP	Cytosol		GFP fusion [15]	yes	yes	no	N	TN	TN	TN
23257	Lhcr11	Pl (thylakoid membrane, PS I)		two-dimensional BN/SDS-PAGE of thylakoid membranes followed by mass spectrometry [13]	yes	yes	yes	P	TP	TP	TP
23414	sTLP-1	BLS		GFP fusion [28]	no	yes	no	N	TN	TN	TN
24610	Tpt1	Pl		GFP fusion [20, 21]	yes	yes	yes	P	TP	FN	TP
25127	NDK3	Mitochondria	protein ID in reference by Moog et al.[28]: 18001	GFP fusion [28]	yes	yes	no	N	TN	TN	TN

Protein ID	Name	Location	Comment	Method	Filtered models?	Optimized gene catalog?	Scoring matrix?	Count	ASAFind, high or low confidence	ASAFind, high confidence only	HECTAR
25168	Lhcf4, FcpD	Pl (thylakoid membrane, FCP trimer)	not used for statistics; full sequence is identical to Lhcf3/-FcpC (in original publication [5], fcpC and fcpD differ slightly from each other, and from the Phatr2 lhcf3/4)	sucrose density gradient centrifugation followed by SDS-PAGE and mass spectrometry [25] + GFP fusion [11] + two-dimensional BN/SDS-PAGE of thylakoid membranes followed by mass spectrometry [13]	*not counted*						
25172	Lhcf2	Pl (thylakoid membrane, FCP trimer)		two-dimensional BN/SDS-PAGE of thylakoid membranes followed by mass spectrometry [13]	yes	yes	no	P	TP	TP	TP
25308	TPI/-GapC3	Mitochondria		immuno electron microscopy with GapC3 antibody [26]	yes	yes	no	N	TN	TN	TN
27278	Lhcx1	Pl (thylakoid membrane, PS I + free protein)	N-terminal extension possible, compare to protein ID 56679	two-dimensional BN/SDS-PAGE of thylakoid membranes followed by mass spectrometry [13]	yes	yes	no	P	TP	TP	TP
29014	FBA3	Cytosol		YFP fusion [2]	yes	yes	no	N	TN	TN	TN
29064	Lhcr13	Pl (thylakoid membrane, PS I)		two-dimensional BN/SDS-PAGE of thylakoid membranes followed by mass spectrometry [13]	no	yes	no	P	TP	TP	FN
30031	Lhcf9	Pl (thylakoid membrane, FCP trimer)		two-dimensional BN/SDS-PAGE of thylakoid membranes followed by mass spectrometry [13]	yes	yes	no	P	TP	TP	FN
30648	Lhcf5/-FcpE	Pl (thylakoid membrane, FCP trimer)		sucrose density gradient centrifugation followed by SDS-PAGE and mass spectrometry [25] + mass spectrometry and western-blot after thylakoid membrane preparation [19] + two-dimensional BN/SDS-PAGE of thylakoid membranes followed by mass spectrometry [13]	yes	yes	no	P	FN	FN	TP
31451	FBPC3	Pl		GFP fusion [15]	no	yes	no	P	FN	FN	TP
31697	Der1-1	BLS		GFP fusion [30, 17]	no	yes	no	N	FP	TN	TN
31704	sORF139	BLS		GFP fusion [28]	yes	yes	no	N	TN	TN	TN
31720	TrxO	Mitochondria		GFP fusion [35]	no	yes	no	N	FP	TN	TN
33356	Trx-y	Pl		GFP fusion [35]	yes	yes	yes	P	TP	TP	FN
34536	Lhcf16	Pl (thylakoid membrane, PS I)		two-dimensional BN/SDS-PAGE of thylakoid membranes followed by mass spectrometry [13]	yes	yes	yes	P	TP	TP	TP

Protein ID	Name	Location	Comment	Method	Filtered models?	Optimized gene catalog?	Scoring matrix?	Count	ASAFind, high or low confidence	ASAFind, high confidence only	HECTAR
34592	CPF2	ER		GFP fusion [28]	yes	yes	no	N	TN	TN	FP
35370	CA-I/-sαCA-1	BLS		GFP fusion [28, 32]	yes	yes	no	N	TN	TN	TN
35695	sDer1-2	BLS		GFP fusion [30, 17]	no	yes	no	N	TN	TN	TN
37614	hDer1-2	ER		GFP fusion [17]	no	yes	no	N	TN	TN	TN
38631	PGL	Pl		GFP fusion [15]	yes	no	yes	P	TP	TP	FN
42406	PtCA1	Pl		GFP fusion [32, 33, 22, 23] + immuno electron microscopy with GFP antibody [32] + CFP fusion [2]	yes	yes	no	P	TP	TP	FN
42447	FBA4	Cytosol		YFP fusion [2]	yes	yes	no	N	TN	TN	TN
42456	FBPC2	Pl		GFP fusion [15]	no	yes	no	P	TP	TP	FN
42543	predicted, PGRL	Pl (thylakoid membrane, free protein)		two-dimensional BN/SDS-PAGE of thylakoid membranes followed by mass spectrometry [13]	yes	yes	no	P	TP	TP	TP
42574	CA-VII/-sαCA-2	ER/BLS	divergent results between the two studies, clearly no plastid protein	GFP fusion [28, 32]	yes	yes	no	N	FP	TN	TN
42675	sSMC	BLS		GFP fusion [28]	yes	yes	no	N	TN	TN	TN
42886	FBPC1	Pl		GFP fusion [15]	no	yes	yes	P	TP	TP	FN
44080	STK4	ER	protein ID in reference by Moog et al.[28]: 33437	GFP fusion [28]	no	yes	no	N	TN	TN	TN
44172	HAP	ER		GFP fusion [28]	yes	yes	no	N	TN	TN	FP
44526	CA-II	BLS	N-terminal extension with predicted mitochondrial transit peptide possible, compare to protein ID 56729	GFP fusion [32]	yes	no	no	N	TN	TN	TN
44601	Lhcr1	Pl (thylakoid membrane, PS I)		two-dimensional BN/SDS-PAGE of thylakoid membranes followed by mass spectrometry [13]	no	yes	no	P	FN	FN	TP
44766	sSec14	BLS		GFP fusion [28]	yes	yes	no	N	TN	TN	TN
44959	sDTC	BLS	protein ID in reference by Moog et al.[28]: 34512	GFP fusion [28]	yes	yes	no	N	FP	TN	TN
45333	PGDH	BLS		GFP fusion [15]	no	yes	no	N	TN	TN	TN

Protein ID	Name	Location	Comment	Method	Filtered models?	Optimized gene catalog?	Scoring matrix?	Count	ASAFind, high or low confidence	ASAFind, high confidence only	HECTAR
45347	sbeta2	BLS	protein ID in reference by Moog et al.[28]: 35028	GFP fusion [28]	no	yes	no	N	FP	TN	TN
45443	PtCa2	Pl		GFP fusion [22]	yes	yes	no	P	TP	FN	FN
45601	SybD	ER		GFP fusion [28]	yes	yes	no	N	TN	TN	TN
45679	unnamed	Extracellular		mass spectrometric peptide mapping of extracellular proteins [7]	yes	yes	no	N	TN	TN	TN
45935	sORF532a	BLS		GFP fusion [28]	yes	yes	no	N	TN	TN	TN
46280	Trx-f	Pl		GFP fusion [35]	no	yes	yes	P	TP	TP	TP
46336	Predicted, low CO_2-inducible	Pl (thylakoid membrane, PS II monomer)		two-dimensional BN/SDS-PAGE of thylakoid membranes followed by mass spectrometry [13]	yes	yes	no	P	TP	TP	TP
46781	PPG1	Pl	protein ID in reference by Moog et al.[28]: 36817	GFP fusion [28]	no	yes	no	P	TP	FN	FN
46875	unnamed	Extracellular		mass spectrometric peptide mapping of extracellular proteins [7]	no	yes	yes	N	FP	TN	TN
47165	unnamed	Extracellular		mass spectrometric peptide mapping of extracellular proteins [7]	yes	yes	no	N	TN	TN	TN
47444	sPUB	BLS	protein ID in reference by Moog et al.[28]: 37661	GFP fusion [28]	no	yes	no	N	TN	TN	TN
47492	Tom70	Mitochondria	protein ID in reference by Moog et al.[28]: 37716	GFP fusion [28]	yes	yes	no	N	TN	TN	TN
47685	FolC	Mitochondria	protein ID in reference by Moog et al.[28]: 37961	GFP fusion [28]	yes	yes	no	N	FP	TN	TN
47766	sP4H	BLS		GFP fusion [28]	yes	yes	no	N	TN	TN	TN
47813	Lhcr14	Pl (thylakoid membrane, PS I)		two-dimensional BN/SDS-PAGE of thylakoid membranes followed by mass spectrometry [13]	no	yes	no	P	TP	TP	TP
48034	ptE3P	BLS		GFP fusion [18]	yes	yes	no	N	TN	TN	TN
48050	Fru	ER	protein ID in reference by Moog et al.[28]: 38415	GFP fusion [28]	no	no	no	N	TN	TN	TN
48220	Sec24-like	Cytosol		GFP fusion [28]	yes	yes	no	N	TN	TN	TN
48423	PtFAD6	Pl		GFP fusion [10]	yes	yes	no	P	TP	FN	FN

Protein ID	Name	Location	Comment	Method	Filtered models?	Optimized gene catalog?	Scoring matrix?	Count	ASAFind, high or low confidence	ASAFind, high confidence only	HECTAR
48518	putative zink transporter	ER		GFP fusion [9]	yes	yes	no	N	TN	TN	TN
48539	TrxH	BLS		GFP fusion [35]	no	yes	no	N	TN	TN	TN
48633	sDPC	BLS		GFP fusion [28]	no	no	no	N	FP	TN	TN
48798	unnamed	Pl (thylakoid membrane, PS I)		two-dimensional BN/SDS-PAGE of thylakoid membranes followed by mass spectrometry [13]	no	yes	no	P	TP	TP	TP
48879	sORF534	BLS		GFP fusion [28]	yes	yes	no	N	TN	TN	TN
49272	unknown protein	ER		GFP fusion [9]	yes	yes	no	N	TN	TN	TN
49432	sbeta6	BLS		GFP fusion [28]	no	yes	no	N	TN	TN	TN
49533	Ntt1	Pl		GFP fusion [4]	yes	yes	yes	P	TP	TP	TP
49840	ORF387	ER		GFP fusion [28]	yes	yes	no	N	FP	TN	FP
49912	Lhcf12	Pl (thylakoid membrane, FCP trimer)		two-dimensional BN/SDS-PAGE of thylakoid membranes followed by mass spectrometry [13]	no	yes	no	P	TP	FN	TP
50476	Glutathione peroxidase	ER		GFP fusion [28]	yes	yes	no	N	TN	TN	TN
50705	Lhcf3, FcpC	Pl (thylakoid membrane, FCP trimer)	identical sequence as Lhcf4/-FcpD (in original publication [5], fcpC and fcpD differ slightly from each other, and from the Phatr2 lhcf3/4)	sucrose density gradient centrifugation followed by SDS-PAGE and mass spectrometry [25] + GFP fusion [11] + two-dimensional BN/SDS-PAGE of thylakoid membranes followed by mass spectrometry [13]	yes	yes	no	P	TP	TP	TP
50725	Lhcr3	Pl (thylakoid membrane, PS I)		two-dimensional BN/SDS-PAGE of thylakoid membranes followed by mass spectrometry [13]	no	yes	no	P	TP	TP	TP
50819	Tkl	Extracellular	plastidic function and prediction result, might be an intracellular contaminant identified in the study by Bruckner et al. [7]	mass spectrometric peptide mapping of extracellular proteins [7]	no	yes	no	N	FP	FP	TN
50907	FtrB	Pl		GFP fusion [35]	no	yes	yes	P	TP	TP	FN
50978	sCdc48	BLS		GFP fusion [30]	no	yes	no	N	TN	TN	TN

Protein ID	Name	Location	Comment	Method	Filtered models?	Optimized gene catalog?	Scoring matrix?	Count	ASAFind, high or low confidence	ASAFind, high confidence only	HECTAR
51055	NTR	Cytosol		GFP fusion [35]	no	yes	no	N	TN	TN	TN
51092	GSII	Pl		YFP fusion [29]	yes	yes	yes	P	TP	TP	TP
51230	Lhcf11	Pl (thylakoid membrane, FCP trimer)	not used for statistics; first 67 residues are identical to Lhcf5/FcpE	two-dimensional BN/SDS-PAGE of thylakoid membranes followed by mass spectrometry [13]				*not counted*			
51289	FbaC5	Pl		YFP fusion and immuno electron microscopy with GFP antibody [2]	no	yes	no	P	TP	TP	TP
51357	Trx-m	Pl		GFP fusion [35]	no	yes	yes	P	TP	TP	TP
53935	Rpe	Pl		GFP fusion [9]	yes	yes	no	P	TP	TP	TP
54013	RecA	Pl		GFP fusion [27]	no	yes	no	P	TP	TP	FN
54027	Lhcr12	Pl (thylakoid membrane, PS I)		two-dimensional BN/SDS-PAGE of thylakoid membranes followed by mass spectrometry [13]	yes	yes	no	P	TP	TP	TP
54246	Bip	ER		GFP fusion [3, 21]	yes	yes	no	N	TN	TN	TN
54251	CA-VI/-αCA-2	ER		GFP fusion [28, 32]	yes	yes	no	N	TN	TN	TN
54279	FBPC4	Pl	in the first study this sequence gave an ambigous result (plastid plus additional compartment?), never any BLS	GFP fusion [15, 14]	no	yes	yes	P	TP	TP	FN
54323	Ubi	BLS		GFP fusion [30]	yes	yes	no	N	TN	TN	TN
54863	TRD1	BLS		GFP fusion [9]	yes	yes	no	N	TN	TN	TN
55029	CA-III	ER		GFP fusion [32]	yes	yes	no	N	FP	TN	TN
55112	Hlip2	Pl		GFP fusion [14]	yes	yes	no	P	TP	TP	FN
55162	Ank5	Mitochondria	protein ID in reference by Moog et al.[28]: 41153	GFP fusion [28]	yes	yes	no	N	TN	TN	TN
55817	unnamed	Extracellular		mass spectrometric peptide mapping of extracellular proteins [7]	no	yes	no	N	TN	TN	TN
55890	Hsp70_2	BLS		GFP fusion [12]	no	yes	no	N	TN	TN	TN
56310	Lhcf17	Pl (thylakoid membrane, PS I)		two-dimensional BN/SDS-PAGE of thylakoid membranes followed by mass spectrometry [13]	no	yes	no	P	TP	TP	TP

Protein ID	Name	Location	Comment	Method	Filtered models?	Optimized gene catalog?	Scoring matrix?	Count	ASAFind, high or low confidence	ASAFind, high confidence only	HECTAR
56467	SBPase	Cytosol		GFP fusion [15]	no	yes	no	N	TN	TN	TN
56471	TrxH	Cytosol		GFP fusion [35]	yes	yes	no	N	TN	TN	TN
56497	GLRX2	BLS		GFP fusion [9]	no	yes	no	N	TN	TN	TN
56519	NTRC	BLS	N-terminal extension possible, compare to protein ID 47568	GFP fusion [35]	no	no	no	N	TN	TN	TN
56618	unCPS	Mitochondria		YFP fusion and immuno electron microscopy with GFP antibody [1]	no	yes	no	N	TN	TN	TN
56648	unknown protein	BLS		GFP fusion [9]	yes	yes	no	N	TN	TN	TN
56658	sDrp (Drp5b)	BLS	protein ID in reference by Moog et al.[28]: 37379, construct tested in [28] is two N-terminal amino acids shorter than this gene model, this is without consequence for the position of the SignalP predicted cleavage site	GFP fusion [28]	no	yes	no	N	FP	TN	TN
56689	Lhcx2	Pl (thylakoid membrane, free protein)		two-dimensional BN/SDS-PAGE of thylakoid membranes followed by mass spectrometry [13]	yes	yes	no	P	TP	FN	TP
56710	ptDUP	BLS		GFP fusion [18]	yes	yes	no	N	TN	TN	TN
56714	unnamed	Extracellular		mass spectrometric peptide mapping of extracellular proteins [7]	yes	yes	no	N	TN	TN	FP
56715	unnamed	Extracellular		mass spectrometric peptide mapping of extracellular proteins [7]	no	no	no	N	TN	TN	TN
56716	unnamed	Extracellular		mass spectrometric peptide mapping of extracellular proteins [7]	no	yes	no	N	TN	TN	TN
56730	salpha7-2	BLS	protein ID in reference by Moog et al.[28]: 43079	GFP fusion [28]	no	no	no	N	TN	TN	TN
56731	sORF261	BLS	protein ID in reference by Moog et al.[28]: 47811	GFP fusion [28]	yes	yes	no	N	TN	TN	TN
56732	Rab1b	ER	protein ID in reference by Moog et al.[28]: 41867	GFP fusion [28]	no	no	no	N	TN	TN	TN

Protein ID	Name	Location	Comment	Method	Filtered models?	Optimized gene catalog?	Scoring matrix?	Count	ASAFind, high or low confidence	ASAFind, high confidence only	HECTAR
56741	ptOmp85	Pl (third outermost envelope membrane)	supposedly enters the third outermost envelope membrane via the stroma (no experimental data on this process available)	GFP fusions of different lengths and self-assembling GFP with Hsp70 [8]	yes	yes	no	P	TP	TP	TP
56747	unnamed	Pl (thylakoid membrane, PS I)		two-dimensional BN/SDS-PAGE of thylakoid membranes followed by mass spectrometry [13]	no	yes	no	P	TP	TP	TP
56752	PGP_2	Mitochondria	protein ID in reference by Moog et al.[28]: 22127	GFP fusion [28]	no	yes	no	N	FP	TN	TN
56754	sPRP	BLS	protein ID in reference by Moog et al.[28]: 41316	GFP fusion [28]	yes	yes	no	N	FP	TN	TN
56757	sPEL	BLS	protein ID in reference by Moog et al.[28]: 37424	GFP fusion [28]	no	yes	no	N	TN	TN	TN
825_bd	FbaC1	Pl	unmapped sequence	GFP fusion [20, 21, 24, 14] + YFP fusion [2]	yes	yes	yes	P	TP	TP	TP
				**Summary:**	yes: 77<br>no: 55	yes: 121<br>no: 11	yes: 19<br>no: 113	P: 55<br>N: 77	TP: 51<br>TN: 63<br>FP: 14<br>FN: 4	TP: 44<br>TN: 76<br>FP: 1<br>FN: 11	TP: 39<br>TN: 72<br>FP: 5<br>FN: 16

References

[1] Allen AE, Dupont CL, Oborník M, Horák A, Nunes-Nesi A, et al. (2011) Evolution and metabolic significance of the urea cycle in photosynthetic diatoms. Nature 473: 203–207.

[2] Allen AE, Moustafa A, Montsant A, Eckert A, Kroth PG, Bowler C (2012) Evolution and Functional Diversification of Fructose Bisphosphate Aldolase Genes in Photosynthetic Marine Diatoms. Molecular Biology and Evolution 29: 367–379.

[3] Apt KE, Zaslavkaia L, Lippmeier JC, Lang M, Kilian O, et al. (2002) *In vivo* characterization of diatom multipartite plastid targeting signals. J Cell Sci 115: 4061–4069.

[4] Ast M, Gruber A, Schmitz-Esser S, Neuhaus HE, Kroth PG, et al. (2009) Diatom plastids depend on nucleotide import from the cytosol. Proc Natl Acad Sci USA 106: 3621–3626.

[5] Bhaya D, Grossman AR (1993) Characterization of gene clusters encoding the fucoxanthin chlorophyll proteins of the diatom *Phaeodactylum tricornutum*. Nucleic Acids Research 21: 4458–4466.

[6] Bowler C, Allen AE, Badger JH, Grimwood J, Jabbari K, et al. (2008) The *Phaeodactylum* genome reveals the evolutionary history of diatom genomes. Nature 456: 239–244.

[7] Bruckner CG, Rehm C, Grossart HP, Kroth PG (2011) Growth and release of extracellular organic compounds by benthic diatoms depend on interactions with bacteria. Environmental Microbiology 13: 1052–1063.

[8] Bullmann L, Haarmann R, Mirus O, Bredemeier R, Hempel F, et al. (2010) Filling the Gap, Evolutionarily Conserved Omp85 in Plastids of Chromalveolates. Journal of Biological Chemistry 285: 6848–6856.

[9] Burmeister C (2009). Lokalisation möglicher periplastidärer Proteine in der Diatomee *Phaeodactylum tricornutum* (in German). Bachelorarbeit, Universität Konstanz.

[10] Domergue F, Spiekermann P, Lerchl J, Beckmann C, Kilian O, et al. (2003) New insight into *Phaeodactylum tricornutum* fatty acid metabolism. Cloning and functional characterization of plastidial and microsomal delta12-fatty acid desaturases. Plant Physiol 131: 1648–1660.

[11] Felsner G, Sommer M, Maier U (2010) The physical and functional borders of transit peptide-like sequences in secondary endosymbionts. BMC Plant Biology 10: 223.

[12] Gould SB, Sommer MS, Kroth PG, Gile GH, Keeling PJ, Maier UG (2006) Nucleus-to-Nucleus Gene Transfer and Protein Retargeting into a Remnant Cytoplasm of Cryptophytes and Diatoms. Mol Biol Evol 23: 2413–2422.

[13] Grouneva I, Rokka A, Aro EM (2011) The Thylakoid Membrane Proteome of Two Marine Diatoms Outlines Both Diatom-Specific and Species-Specific Features of the Photosynthetic Machinery. Journal of Proteome Research 10: 5338–5353.

[14] Gruber A, Vugrinec S, Hempel F, Gould S, Maier UG, Kroth P (2007) Protein targeting into complex diatom plastids: functional characterisation of a specific targeting motif. Plant Mol Biol 64: 519–530.

[15] Gruber A, Weber T, Río Bártulos C, Vugrinec S, Kroth PG (2009) Intracellular distribution of the reductive and oxidative pentose phosphate pathways in two diatoms. Journal of Basic Microbiology 49: 58–72.

[16] Gschloessl B, Guermeur Y, Cock JM (2008) HECTAR: A method to predict subcellular targeting in heterokonts. BMC Bioinformatics 9: 393.

[17] Hempel F, Bullmann L, Lau J, Zauner S, Maier UG (2009) ERAD-Derived Preprotein Transport across the Second Outermost Plastid Membrane of Diatoms. Molecular Biology and Evolution 26: 1781–1790.

[18] Hempel F, Felsner G, Maier UG (2010) New mechanistic insights into preprotein transport across the second outermost plastid membrane of diatoms. Molecular Microbiology 76: 793–801.

[19] Joshi-Deo J, Schmidt M, Gruber A, Weisheit W, Mittag M, et al. (2010) Characterization of a trimeric light-harvesting complex in the diatom *Phaeodactylum tricornutum* built of FcpA and FcpE proteins. Journal of Experimental Botany 61: 3079–3087.

[20] Kilian O, Kroth PG (2004) Presequence acquisition during secondary endocytobiosis and the possible role of introns. J Mol Evol 58: 712–721.

[21] Kilian O, Kroth PG (2005) Identification and characterization of a new conserved motif within the presequence of proteins targeted into complex diatom plastids. Plant J 41: 175–183.

[22] Kitao Y, Harada H, Matsuda Y (2008) Localization and targeting mechanisms of two chloroplastic β-carbonic anhydrases in the marine diatom *Phaeodactylum tricornutum*. Physiologia Plantarum 133: 68–77.

[23] Kitao Y, Matsuda Y (2009) Formation of macromolecular complexes of carbonic anhydrases in the chloroplast of a marine diatom by the action of the C-terminal helix. Biochem J 419: 681–688.

[24] Kroth PG, Schroers Y, Kilian O (2005) The peculiar distribution of class I and class II aldolases in diatoms and in red algae. Curr Genet 48: 389–400.

[25] Lepetit B, Volke D, Szabó M, Hoffmann R, Garab G, et al. (2007) Spectroscopic and Molecular Characterization of the Oligomeric Antenna of the Diatom *Phaeodactylum tricornutum*. Biochemistry 46: 9813–9822.

[26] Liaud M, Lichtle C, Apt K, Martin W, Cerff R (2000) Compartment-specific isoforms of TPI and GAPDH are imported into diatom mitochondria as a fusion protein: Evidence in favor of a mitochondrial origin of the eukaryotic glycolytic pathway. Mol Biol Evol 17: 213–223.

[27] Materna A (2006). Development of molecular tools in the diatom *Phaeodactylum tricornutum*. Dissertation, Universität Konstanz.

[28] Moog D, Stork S, Zauner S, Maier UG (2011) In Silico and In Vivo Investigations of Proteins of a Minimized Eukaryotic Cytoplasm. Genome Biology and Evolution 3: 375–382.

[29] Siaut M, Heijde M, Mangogna M, Montsant A, Coesel S, et al. (2007) Molecular toolbox for studying diatom biology in *Phaeodactylum tricornutum*. Gene 406: 23–35.

[30] Sommer MS, Gould SB, Lehmann P, Gruber A, Przyborski JM, Maier UG (2007) Der1-mediated Preprotein Import into the Periplastid Compartment of Chromalveolates? Mol Biol Evol 24: 918–928.

[31] Sturm S, Engelken J, Gruber A, Vugrinec S, Kroth PG, et al. (2013) A novel type of light-harvesting antenna protein of red algal origin in algae with secondary plastids. BMC Evolutionary Biology 13: 159.

[32] Tachibana M, Allen A, Kikutani S, Endo Y, Bowler C, Matsuda Y (2011) Localization of putative carbonic anhydrases in two marine diatoms, *Phaeodactylum tricornutum* and *Thalassiosira pseudonana*. Photosynthesis Research 109: 205–221. ISSN 0166-8595.

[33] Tanaka Y, Nakatsuma D, Harada H, Ishida M, Matsuda Y (2005) Localization of Soluble β-Carbonic Anhydrase in the Marine Diatom *Phaeodactylum tricornutum*. Sorting to the Chloroplast and Cluster Formation on the Girdle Lamellae. Plant Physiology 138: 207–217.

[34] Vugrinec S, Gruber A, Kroth PG (2011) Protein targeting into complex plastids—support for the translocator model. Endocytobiosis and Cell Research 21: 59–63.

[35] Weber T, Gruber A, Kroth PG (2009) The Presence and Localization of Thioredoxins in Diatoms, Unicellular Algae of Secondary Endosymbiotic Origin. Molecular Plant 2: 468–477.
