# Supplementary material for: Plastid proteome prediction for diatoms and other algae with secondary plastids of the red lineage
Source: Plant J. 2015 Jan 6;81(3):519–28. doi: 10.1111/tpj.12734 (PMC4329603; doi:10.1111/tpj.12734)
Supplement: Table S6 — Prediction statistics. [file tpj0081-0519-sd8.pdf]

Table S6[1]: Prediction statistics. Statistical evaluation of diatom plastid protein prediction, see text for details on the reference sets and prediction methods, numbers in parentheses are counts/scores without those sequences which have been used to calculate the scoring matrix (Table S1). FN, false negative; FP, false positive; N, negative; P, positive; TN, true negative; TP, true positive.

		Reference set	Experimentally determined locations (Tables S3, S4)			Mutated sequences (Table S5)		
Positives	P		55 (37)			22		
Negatives	N		77 (76)			27		
Total number	$P+N$		132 (113)			49		
		Prediction method	ASAFind, high confidence only[1]	ASAFind, high or low confidence[1]	HECTAR[2]	ASAFind, high confidence only[1]	ASAFind, high or low confidence[1]	HECTAR[2]
True positives	TP		44 (28)	51 (34)	39 (27)	11	19	14
True negatives	TN		76 (75)	63 (63)	72 (71)	18	10	19
False positives	FP		1 (1)	14 (13)	5 (5)	9	17	8
False negatives	FN		11 (9)	4 (3)	16 (10)	11	3	8
Sensitivity (true positive rate, recall)	$\frac{TP}{P}$		0.80 (0.76)	0.93 (0.92)	0.71 (0.73)	0.50	0.86	0.64
Specificity (true negative rate)	$\frac{TN}{N}$		0.99 (0.99)	0.82 (0.83)	0.94 (0.93)	0.67	0.37	0.70
False positive rate	$\frac{FP}{N}$		0.01 (0.01)	0.18 (0.17)	0.06 (0.07)	0.33	0.63	0.30
False negative rate	$\frac{FN}{P}$		0.20 (0.24)	0.07 (0.08)	0.29 (0.27)	0.50	0.14	0.36
Accuracy	$\frac{TP+TN}{P+N}$		0.91 (0.91)	0.86 (0.86)	0.84 (0.87)	0.59	0.59	0.67
Positive predictive value (precision)	$\frac{TP}{TP+FP}$		0.98 (0.97)	0.78 (0.72)	0.89 (0.84)	0.55	0.53	0.64
Negative predictive value	$\frac{TN}{TN+FN}$		0.87 (0.89)	0.94 (0.95)	0.82 (0.88)	0.62	0.77	0.70
False discovery rate	$\frac{FP}{FP+TP}$		0.02 (0.03)	0.22 (0.28)	0.11 (0.16)	0.45	0.47	0.36
Matthews correlation coefficient	$\frac{TP\times TN-FP\times FN}{\sqrt{(TP+FP)\times(TP+FN)\times(TN+FP)\times(TN+FN)}}$		0.82 (0.80)	0.74 (0.71)	0.67 (0.69)	0.17	0.26	0.34

[1]Gruber *et al.*, doi: 10.1111/tpj.12734
[2]Gschloessl *et al.*, doi: 10.1186/1471-2105-9-393
