# Supplementary material for: Plastid proteome prediction for diatoms and other algae with secondary plastids of the red lineage
Source: Plant J. 2015 Jan 6;81(3):519–28. doi: 10.1111/tpj.12734 (PMC4329603; doi:10.1111/tpj.12734)
Supplement: Supplementary file 17 [file tpj0081-0519-sd17.pdf]

Legends for Supplementary Material (Gruber *et al.*, http://dx.doi.org/10.1111/tpj.12734)

Figure S1: Conserved transit peptides. Conserved N-termini of transit peptide domains of bipartite diatom plastid targeting pre-sequences downstream of the signal peptide cleavage site. Sequence logos and frequency plots (Schneider and Stephens 1990) for 83 putative plastid-targeted protein sequences from *Thalassiosira pseudonana* and *Phaeodactylum tricornutum* (Table S1) and for the transit peptides published by Huesgen *et al.* (Table S7 of Huesgen *et al.* (2013)). Sequence logos and frequency plots created with WebLogo (http://weblogo.berkeley.edu/) (Crooks *et al.* 2004). (PDF file)

Figure S2: Cleavage site motif identification. Number of cleavage site motif identifications by the different versions of SignalP (Bendtsen *et al.* 2004, Emanuelsson *et al.* 2007, Nielsen and Krogh 1998, Petersen *et al.* 2011) compared to manually identified motifs (see also Table S1), 83 sequences per organism, 166 sequences in the combined set. (PDF file)

Table S1: Initial Sequence Set. 166 putative plastid-targeted protein sequences from *T. pseudonana* and *P. tricornutum* used to construct the scoring matrix for plastid targeting motifs, sequence identifiers, manually identified cleavage site motifs, prediction results of the different SignalP versions (Bendtsen*, et al.* 2004, Emanuelsson*, et al.* 2007, Nielsen and Krogh 1998, Petersen*, et al.* 2011) and matches of N-TAILS peptides identified by Huesgen *et al.* (Table S5 of Huesgen*, et al.* (2013)) are listed as indicated. (Text file, tab-separated values)

Table S2: Scoring Matrix. Scoring matrix based on sequence logos around the SignalP cleavage site for 83 orthologous pairs of putative plastid-targeted protein sequences from *T. pseudonana* and *P. tricornutum* (see Figure 1, Table S1). Scores are calculated using the frequency of occurrence of each amino acid weighted by the amount of information at each position (Schneider and Stephens 1990). (Text file, tab-separated values)

Table S3: Reference set. Proteins with experimentally determined intracellular location in *Phaeodactylum tricornutum*. Protein IDs refer to the U.S. Department of Energy Joint Genome Institute *Phaeodactylum tricornutum* v2.0 database (http://genome.jgi-psf.org/Phatr2/Phatr2.home.html) (Bowler *et al.* 2008); the 'Filtered models?' column indicates whether the filtered models contain a sequence with identical N-terminus (first 60 residues); the 'Optimized gene catalog?' column indicates whether the optimized gene catalog contains a sequence with identical N-terminus (first 60 residues); the 'Scoring matrix?' column indicates whether a sequence was used to build the matrix used to calculate the plastid score; 'Count' indicates whether the sequence was counted as positive or negative; the 'ASAFind, high or low confidence', 'ASAFind, high confidence only' and 'HECTAR' (Gschloessl *et al.* 2008) columns indicate the classification of the sequence with each prediction method, see text for details. BLS, 'blob'-like structure (Kilian and Kroth 2005); FCP, fucoxanthin chlorophyll a/c-binding protein); FN, false negative; FP, false positive; ies, inter envelope space (compartment between the innermost and second innermost plastid membranes; N, negative; P, positive; Pl, plastid; PS, Photosystem; TN, true negative; TP, true positive. (PDF file)

Table S4: Prediction results for native reference sequences. Detailed plastid protein prediction results for the evaluation set sequences listed in Table S1. Protein IDs refer to the U.S. Department of Energy Joint Genome Institute *P. tricornutum* v2.0 database (http://genome.jgi-psf.org/Phatr2/Phatr2.home.html) (Bowler*, et al.* 2008); 'name', 'location' and 'count' re-print the results of the experimental studies (see legend of Table S3 for details and abbreviations); the 'ASAFind, high or low confidence', 'ASAFind, high confidence only' and 'HECTAR' columns indicate the classification of the sequence with each prediction method (see text and legend of Table S3 for details and abbreviations); the 'SignalP' column indicates whether a signal peptide was identified by SignalP 3.0 NN; the 'SignalP D score' reprints the D score generated by SignalP for the signal peptide prediction; 'Signal P cleavage position' is the cleavage site position predicted by SignalP 3.0 NN; 'SignalP 25aa cleavage score' is the

score of the 25 position sequence window at the SignalP predicted cleavage site; 'SignalP 25aa sequence' is the 25 position sequence window at the SignalP predicted cleavage site; the 'ASAFind cleavage position' is the cleavage position resulting in the highest scoring sequence window surrounding the SignalP predicted cleavage site; 'ASAFind 25aa cleavage score' is the score of the 25 position sequence window at the ASAFind predicted cleavage site; 'ASAFind 25aa sequence' is the 25 position sequence window at the ASAFind predicted cleavage site; 'ASAFind cleavage has [FWYL] at +1 position' indicates whether the +1 position of the best scoring sequence window is either 'F', 'W', 'Y' or 'L'; 'ASAFind/SignalP cleavage site offset' is the offset between the best scoring sequence window and the cleavage site position predicted by SignalP; 'ASAFind 20aa transit score' is the score of the 20 position sequence window after the ASAFind predicted cleavage site; 'ASAFind prediction' is the resulting plastid protein prediction; 'HECTAR predicted subcellular localization' and following two columns contain the HECTAR prediction result for the sequence, see Gschloessl*, et al.* (2008) for details. NA, not applicable. (Text file, tab-separated values)

Table S5: Prediction results for mutated reference sequences. Detailed plastid protein prediction results for the mutated plastid targeting pre-sequences investigated by Felsner *et al.* (2010), Gruber *et al.* (2007) and Kilian and Kroth (2005). See caption of Table S4 for column header descriptions and abbreviations (Text file, tab-separated values)

Table S6: Prediction statistics. Statistical evaluation of diatom plastid protein prediction, see text for details on the reference sets and prediction methods, numbers in parentheses are counts/scores without those sequences which have been used to calculate the scoring matrix (Table S1). FN, false negative; FP, false positive; N, negative; P, positive; TN, true negative; TP, true positive. (PDF file)

Table S7: Prediction results for *Thalassiosira pseudonana*. Detailed plastid protein prediction results for the optimized *Thalassiosira pseudonana* gene catalog (Dataset S1). Identifiers are protein IDs as

described in Dataset S1; the ‘SignalP’ column indicates whether a signal peptide was identified by SignalP 3.0 NN; the ‘SignalP D score’ reprints the D score generated by SignalP for the signal peptide prediction; ‘Signal P cleavage position’ is the cleavage site position predicted by SignalP 3.0 NN; ‘SignalP 25aa cleavage score’ is the score of the 25 position sequence window at the SignalP predicted cleavage site; ‘SignalP 25aa sequence’ is the 25 position sequence window at the SignalP predicted cleavage site; the ‘ASAFind cleavage position’ is the cleavage position resulting in the highest scoring sequence window surrounding the SignalP predicted cleavage site; ‘ASAFind 25aa cleavage score’ is the score of the 25 position sequence window at the ASAFind predicted cleavage site; ‘ASAFind 25aa sequence’ is the 25 position sequence window at the ASAFind predicted cleavage site; ‘ASAFind cleavage has [FWYL] at +1 position’ indicates whether the +1 position of the best scoring sequence window is either ‘F’, ‘W’, ‘Y’ or ‘L’; ‘ASAFind/SignalP cleavage site offset’ is the offset between the best scoring sequence window and the cleavage site position predicted by SignalP; ‘ASAFind 20aa transit score’ is the score of the 20 position sequence window after the ASAFind predicted cleavage site; ‘ASAFind prediction’ is the resulting plastid protein prediction; ‘Protein sequence’ is the protein sequence of the gene model. NA, not applicable. (Text file, tab-separated values)

Table S8: Prediction results for *Phaeodactylum tricornutum*. Detailed plastid protein prediction results for the optimized *Phaeodactylum tricornutum* gene catalog (Dataset S2). Identifiers are protein IDs as described in Dataset S2, see caption of Table S7 for remaining column header descriptions. (Text file, tab-separated values)

Dataset S1: Optimized *Thalassiosira pseudonana* gene catalog. Results of the gene catalog optimization for *Thalassiosira pseudonana*, FASTA descriptions consist of organism (‘Thaps3’), database (‘a’ for assembly or ‘u’ for unmapped, see http://genome.jgi-psf.org/Thaps3/Thaps3.home.html for explanations) and protein IDs, separated by ‘_’ if multiple protein IDs refer to gene models with identical protein sequence. (Text file, FASTA format)

Dataset S2: Optimized *Phaeodactylum tricornutum* gene catalog. Results of the gene catalog optimization for *Phaeodactylum tricornutum*, FASTA descriptions consist of organism ('Phatr2'), database ('a' for assembly' or 'u' for unmapped, see http://genome.jgi-psf.org/Phatr2/Phatr2.home.html for explanations) and protein IDs, separated by '_' if multiple protein IDs refer to gene models with identical protein sequence. (Text file, FASTA format)

Appendix S1: ASAFind python script. ASAFind.py requires Python 2.7 (https://www.python.org/) with Biopython (Cock *et al.* 2009). Takes a FASTA file and a companion SignalP (Bendtsen, *et al.* 2004, Emanuelsson, *et al.* 2007, Petersen, *et al.* 2011) output table in short format (versions 3.0, 4.0 or 4.1, http://www.cbs.dtu.dk/services/SignalP/) as input. The FASTA names and SignalP names must not be longer than 20 characters, be unique within each file and match perfectly between the two files. Call with the '-h' option for help. (Text file, Python syntax)

References

Bendtsen, J.D., Nielsen, H., von Heijne, G. and Brunak, S. (2004) Improved prediction of signal peptides: SignalP 3.0. *J Mol Biol*, **340**, 783-795.

Bowler, C., Allen, A.E., Badger, J.H., Grimwood, J., Jabbari, K., Kuo, A., Maheswari, U., Martens, C., Maumus, F., Otillar, R.P., Rayko, E., Salamov, A., Vandepoele, K., Beszteri, B., Gruber, A., Heijde, M., Katinka, M., Mock, T., Valentin, K., Verret, F., Berges, J.A., Brownlee, C., Cadoret, J.P., Chiovitti, A., Choi, C.J., Coesel, S., De Martino, A., Detter, J.C., Durkin, C., Falciatore, A., Fournet, J., Haruta, M., Huysman, M.J., Jenkins, B.D., Jiroutova, K., Jorgensen, R.E., Joubert, Y., Kaplan, A., Kroger, N., Kroth, P.G., La Roche, J., Lindquist, E., Lommer, M., Martin-Jezequel, V., Lopez, P.J., Lucas, S., Mangogna, M., McGinnis, K., Medlin, L.K., Montsant, A., Oudot-Le Secq, M.P., Napoli, C., Obornik, M., Parker, M.S., Petit, J.L., Porcel, B.M., Poulsen, N., Robison, M., Rychlewski, L., Rynearson, T.A., Schmutz, J., Shapiro, H., Siaut, M., Stanley, M., Sussman, M.R., Taylor, A.R., Vardi, A., von Dassow, P., Vyverman, W., Willis, A., Wyrwicz, L.S., Rokhsar, D.S., Weissenbach, J., Armbrust, E.V., Green, B.R., Van de Peer, Y. and Grigoriev, I.V. (2008) The Phaeodactylum genome reveals the evolutionary history of diatom genomes. *Nature*, **456**, 239-244.

Cock, P.J., Antao, T., Chang, J.T., Chapman, B.A., Cox, C.J., Dalke, A., Friedberg, I., Hamelryck, T., Kauff, F., Wilczynski, B. and de Hoon, M.J. (2009) Biopython: freely available Python tools for computational molecular biology and bioinformatics. *Bioinformatics*, **25**, 1422-1423.

Crooks, G.E., Hon, G., Chandonia, J.M. and Brenner, S.E. (2004) WebLogo: a sequence logo generator. *Genome research*, **14**, 1188-1190.

Emanuelsson, O., Brunak, S., von Heijne, G. and Nielsen, H. (2007) Locating proteins in the cell using TargetP, SignalP and related tools. *Nat Protoc*, **2**, 953-971.

Felsner, G., Sommer, M.S. and Maier, U.G. (2010) The physical and functional borders of transit peptide-like sequences in secondary endosymbionts. *BMC plant biology*, **10**, 223.

Gruber, A., Vugrinec, S., Hempel, F., Gould, S.B., Maier, U.G. and Kroth, P.G. (2007) Protein targeting into complex diatom plastids: functional characterisation of a specific targeting motif. *Plant Mol.Biol.*, **64**, 519-530.

Gschloessl, B., Guermeur, Y. and Cock, J.M. (2008) HECTAR: a method to predict subcellular targeting in heterokonts. *BMC bioinformatics*, **9**, 393.

Huesgen, P.F., Alami, M., Lange, P.F., Foster, L.J., Schroder, W.P., Overall, C.M. and Green, B.R. (2013) Proteomic amino-termini profiling reveals targeting information for protein import into complex plastids. *PLoS One*, **8**, e74483.

Kilian, O. and Kroth, P.G. (2005) Identification and characterization of a new conserved motif within the presequence of proteins targeted into complex diatom plastids. *Plant J*, **41**, 175-183.

Nielsen, H. and Krogh, A. (1998) Prediction of signal peptides and signal anchors by a hidden Markov model. *Proceedings / ... International Conference on Intelligent Systems for Molecular Biology ; ISMB. International Conference on Intelligent Systems for Molecular Biology*, **6**, 122-130.

Petersen, T., Brunak, S., von Heijne, G. and Nielsen, H. (2011) Signal1 4.01 discriminating signal peptides from transmembrane regions. *Nature Methods*, **8**, 785-786.

Schneider, T.D. and Stephens, R.M. (1990) Sequence logos: a new way to display consensus sequences. *Nucleic acids research*, **18**, 6097-6100.
